# Trend dynamics of thyroid cancer incidence among China and the U.S. adult population from 1990 to 2017: a joinpoint and age-period-cohort analysis

**DOI:** 10.1186/s12889-021-10635-w

**Published:** 2021-03-31

**Authors:** Yiran Cui, Sumaira Mubarik, Ruijia Li, Nawsherwan, Chuanhua Yu

**Affiliations:** 1 Department of Epidemiology and Biostatistics, School of Health Sciences, Wuhan University, 185 Donghu Road, Wuhan, 430071 China; 2 Global Health Institute, Wuhan University, Wuhan, 430071 China; 3 Department of Preventive Medicine, School of Health Sciences, Wuhan University, Wuhan, Hubei China

**Keywords:** Incidence, Thyroid cancer, Joinpoint regression analysis, Age-period-cohort effect

## Abstract

**Background:**

Thyroid cancer (TC) is the most common malignant disease of the endocrine system. Based on the previously published reports, the incidence of TC has been increasing in the past 25 years, and the reason for the increase is not yet clear. The present study aims to reveal the long-term trends and age–period–cohort effects for the incidence of TC in China and the U.S. from 1990 to 2017.

**Methods:**

We examined the trends of TC incidence and the average annual percentage change (AAPC) of rate using the Joinpoint regression analysis in the two countries, for the different genders (men/women) in the Global Burden of Disease (GBD 2017). We further used an age-period-cohort model to analyze age-period-cohort effects on TC incidence.

**Results:**

The ASIR of China increased markedly with AAPC of 4.5% (95% confidence interval (CI): 4.0, 5.0%) and 1.8% (1.6, 2.0%) for men and women during 1990–2017. The ASIR of the U. S increased by 1.4% (1.0, 1.8%) and 1.3% (0.9, 1.7%) for men and women from 1990 to 2017.TC increased with the age and period. Aging was one of the most influential factors of TC in China. The age effect increased markedly in the U.S. compared with China. The period effect showed an increase in China while that tended to grow steadily during 1990–2017 in the U.S. The cohort effect peaked in 1963–1967 birth cohorts for men and women in China and declined consistently in the birth cohort in the U.S.

**Conclusion:**

From 1990 to 2017, due to ionizing radiation and over-diagnosis, age-standardized TC incidence rates in both genders rose in China and the U.S. The standardized incidence rate of women is higher than that of men. It is necessary to provide women with reasonable prevention and protection measures for TC. We need to apply for health services and screening to reduce ionizing radiation.

## Background

Due to the rapid increase in the incidence of thyroid cancer (TC), TC is now considered as a major public health problem worldwide [1–3]. From a clinical perspective, TC is also a malignant tumor caused by follicular or parathyroid thyroid cells. In the recent past decades, the incidence of TC has been steadily increasing globally, especially among women [3–7]. Previous studies also indicated a significant increase in the incidence of TC in the Chinese population [8]. The incidence of TC in women is higher (3.8 per 100,000) than that in Chinese men (1.1 per 100,000). This difference may be attributed to female reproductive hormones. It is speculated that the increased levels of the female hormone caused by reproductive events trigger the thyroid hormone levels, which can cause thyroid dysplasia and eventually lead to cancer outbreaks [9]. In many middle and high-income countries, the incidence of TC has been growing rapidly, the incidence of thyroid cancer has risen sharply in the U. S, from 3.6 cases per 100,000 in 1973 to 15 cases per 100,000 in 2014. From a previous study, about 62,450 new cases of TC are reported by the American Cancer Society [10]. In the U.S., women have doubled or tripled in just a few decades. According to some reports, the recent incidence has increased slightly in the U.S. [6, 11]. Meanwhile, the U.S. is one of the representative countries with a high TC incidence. In the U.S. and China, the incidence of TC varies greatly globally, and there is the largest difference between urban and rural areas in China [12].

The 2015 annual report of the Chinese Cancer Registry shows that TC has become one of the top ten cancers that seriously threaten the health of Chinese residents, and it is particularly serious in some provinces such as Zhejiang, with women ranking the first. Therefore, we choose two representative countries to describe the trend of the TC in order to further control and study the incidence of TC [13].

Many studies have different opinions about the influence of age, period and cohort on the incidence of TC. It is believed that the increase in the incidence of TC may be due to over diagnosis [14, 15]. However, environmental factors may lead to an increase in the incidence of TC [16, 17]. In order to assess the trend dynamic of TC incidence due to underlying reasons, we analyzed the temporal changes of TC from 1990 to 2017, stratified by sex and age group using Joinpoint and age-period-cohort model in China and the U.S. population.

## Methods

### Data sources

Thyroid cancer incidence (1990–2017) can be obtained from the Global Health Data Exchange (GHDx) website of the Institute for health metrics and evaluation (IHME). IHME is an independent global health research center at the University of Washington. The incidence rate of TC [International Classification of Diseases (ICD)-10 code] from GBD 2017 provides a comprehensive upshot for the 354 causes in 195 countries and territories from 1990 to 2017 [18]. GBD 2017 estimated annual results for metrics of incidence, prevalence, death, Years of Life Lost (YLLs), Years Lived with Disability (YLDs), and Disability Adjusted Life Years (DALYs) from 1990 to 2017. The GBD study includes the burden of disease in the global population of all age groups, different causes, different regions and different genders [19]. The process for non-fatal estimation starts at the compilation data sources from multiple possible sources, including 21 possible data types of Global Health Data Exchange (GHDx), from scientific literature to survey data to epidemiological surveillance data. In China and the U.S., the original data are not only collected from the literature, but also the vital registry, the Cancer Registry of the Ministry of Health; and the World Health Organization (WHO) mortality database. In every type of cancer for the world population, age is a key determinant of developing the risk of the disease. In order to compare the incidence of multiple populations or time points, we need a summary measure that absorbs the schedule of age-specific incidence in different year. The age-standardized incidence is in an age group with a certain reference population (usually called the standard population). Age-standardized incidence rate (ASIR) from TC was based on the GBD 2017 global age. TC data were analyzed by 5-year periods from 1992 to 2017 and 12 age groups (ranging from 20 to 79 years). The data collection process and the estimation process have been described in detail previously. Therefore, the data quality is very reliable due to strict inspection and verification by various departments.

### Statistical analysis

#### Joinpoint regression analysis

Determining trend changes are an important issue in analyzing cancer mortality and morbidity data. We apply a Joinpoint regression model to describe this continuous change [20]. In this analysis, it was possible to determine when a significant change was detected in the linear slopes of TC incidence trends in China and the U.S. during the study period from 1990 to 2017. The best-fitting points are called the “Joinpoints” (*p* < 0.05). At each Joinpoint, the trend change in the ASIR of TC is observed [21]. We estimated the annual percentage change (APC), the average annual percentage change (AAPC), and the 95% confidence interval (CI) for each segment were estimated for each segment identified by the mode. We also used Monte Carlo methods to find each *p*-value and maintain the overall asymptotic significance level through Bonferroni correction. At the same time, considering that the change in the International Classification of Diseases (ICD) from ICD-9 to ICD-10 may affect the trend of TC incidence in the U.S. and China, I also used the Jump model in their Joinpoint regression analysis [22]. This model is used by many cancer registries around the world for trends in cancer rates [23]. This analysis was conducted using the Joinpoint regression program version 4.6.0.0 (April 2018) from the Surveillance Research Program of the U.S. National Cancer Institute.

#### Age–period–cohort analysis

APC analysis was used to decompose the three trends and provides relatively efficient estimation results [24]. In this analysis, age reflects changes in vital rates, the risk of incidence increases with the age groups. Period effect represents influencing factors, including a series of historical events and environmental factors. Exposure to risk factors is different in different generations. Cohort effects show variations across groups of individuals born during the same period and changes in different lifestyles.

The general form of APC is written as

1 $$ \mathrm{Y}=\log \left(\mathrm{M}\right)=\upmu +{\upalpha \mathrm{age}}_1+{\upbeta \mathrm{period}}_1+{\upgamma \mathrm{cohort}}_1+\upvarepsilon, $$

where, M is defined as the incidence of the age group, α, β, and γ represent the functions of age, period and cohort effect, μ and ε are the intercept item and the random error.

Collinearity is a common problem in the application of APC models, it has age_1_ = period_1_ – cohort_1_. The APC model is affected by the linearity between the two, so it is impossible to determine the three independent linear APC variables of age, period, and cohort. Now, the APC model is dedicated to the innovation of the traditional linear regression model: the intrinsic estimator (IE) is also a new method of coefficient estimation. In our study, APC model with an IE method was used to solve the multicollinearity problem that has been used in various other epidemiological studies [25, 26]. The IE method represents the APC model in the following matrix form:

2 $$ \mathrm{Y}=\mathrm{Xb}+\upvarepsilon $$

where Y denotes the logarithm of TC incidence divided by age, period, and birth cohort; X represents the design matrix; and b is a vector composed of the age, period, and cohort effect coefficients. In the APC model using the IE method, the age-specific rate was appropriately categorized into a continuous 5-year-old age group (20–24, 25–29, 30–34, 35–39, 40–44, 45–49, 50–54, 55–59, 60–64, 65–69, 70–74, and 75–79 years). It has 5-year intervals of periods (1992–1997, 1997–2002, 2002–2007, 2007–2012, and 2012–2017) and 18 cohorts of birth (i.e., 1913–1917,1918–1922, …, 1988–1992,1993–1997).

The APC analysis was conducted using Stata 15.0 software (College Station, TX, USA). Furthermore, a Wald test was carried out peculiarly based on the outcomes of the APC model, due to the corresponding value of *P* < 0.05, which represents a vivid significance statistically. Moreover, the Akaike information criterion (AIC), Bayesian information criterion (BIC), and Deviance were used to estimate and analyze the degree of fitting of the model.

## Results

### Descriptive analysis of TC incidence in China and the U.S

**Fig. 1:**
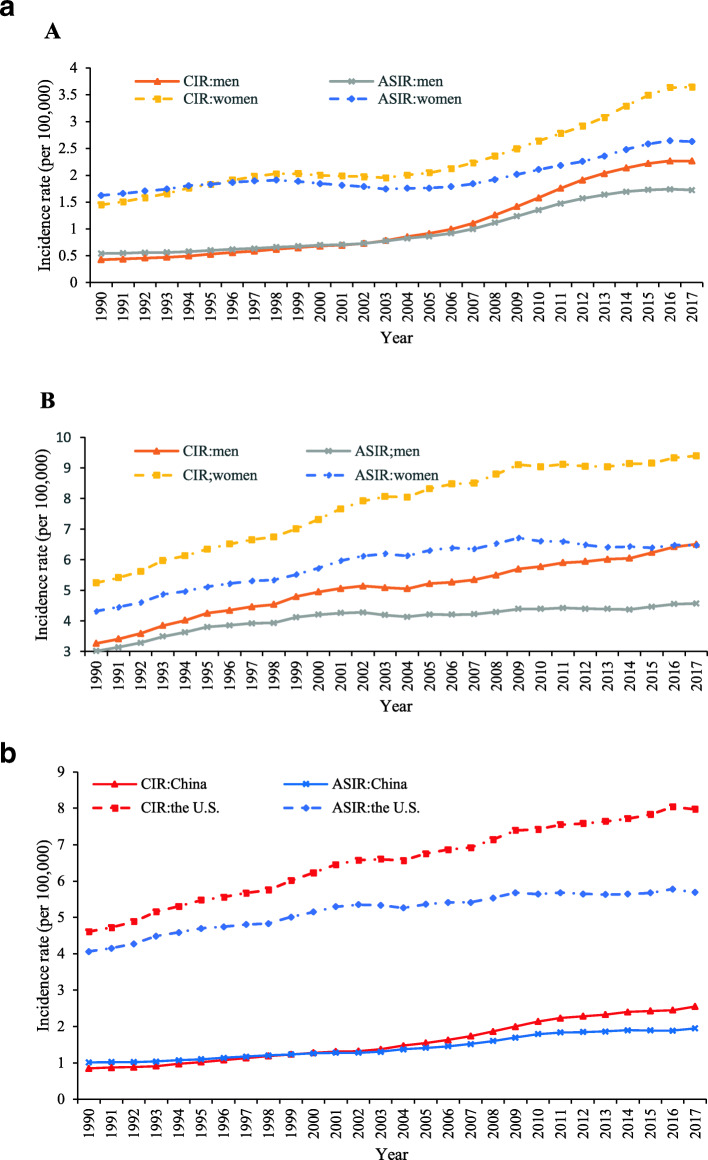
**a**. Trends of the crude incidence rates (CIR) and age-standardized incidence rates (ASIR) for thyroid cancer (TC) in men and women from 1990 to 2017 in (A) China and (B) the U.S. **b**. Trends of the crude incidence rates (CIR) and age-standardized incidence rates (ASIR) for thyroid cancer (TC) in both sexes from 1990 to 2017 in China and the U.S

The trends of the crude incidence rate (CIR), age-standardized incidence rate (ASIR) at all ages from 1990 to 2017 for TC in China and the U. S were presented in Fig. 1a. CIR for men and women increased from 1990 to 2017 in China and the U.S. The ASIR in men increased from 0.54 in 1992 to 1.72 in 2017 per 100,000 persons, and the ASIR in women increased slowly from 1.62 to 2.63 per 100,000 during the same period in China. Overall, in both populations (China and the U.S), compared with men, the higher CIR and ASIR of women have been observed from 1990 to 2017. Among the ASIR of the U.S. increased before 2009 and declined thereafter for TC incidence trend. The trends of CIR and ASIR for TC in both sexes from 1990 to 2017 in China and the U.S. are shown in Fig. 1b.

### Temporal trends of TC incidence in China and the U.S

APC and AAPC of TC incidence by gender from 1990 to 2017 in China and the U.S. are reported in Table 1. JP-Jump models showed a significant increasing trend in China between 1990 and 2017. This trend displayed from 1990 to 2017 in both men and women, with overall AAPC values of 4.5 (4.0, 5.0%) for men and 1.8 (1.6, 2.0%) for women. Moreover, since 1990 the ASIR of TC among Chinese men started to increase significantly and surprisingly, there was a marked increase rate during 2006 to 2011. The comparability ratio (CR) for Chinese men and women is 1.01(0.9, 1.03) and 0.97(0.96, 0.99), with SE =0.011 and 0.007. In the U.S., JP-Jump model results showed that the ASIR of TC among U.S. men increased obviously and then declined t during 2001–2004. However, an increasing trend in ASIR of TC was observed from 1990 to 2009 and then declined during 2009–2017 in the U.S. women. The CR for U.S. men and women is 1.03(0.99, 1.07) and 0.97(0.96, 1.02), with SE =0.017 and 0.015.

**Table 1:** Trends in age-standardized incidence rates of thyroid cancer by gender in China and the U.S., 1990–2017

Jump model	Men		Women		Both sexes	
	Year	APC*(95%CI)	Year	APC * (95% CI)	Year	APC * (95% CI)
China						
Trend1	1990–2002	2.7*(2.4,2.9)	1990–1997	2.4*(2.2,2.6)	1990–1993	0.8(− 2.3,2.9)
Trend2	2002–2006	5.7* (4.3,7.2)	1995–2001	−1.8*(− 2.1,-1.5)	1993–2000	2.7*(2.6,3.3)
Trend3	2006–2011	10.2* (9.3,11.2)	2001–2004	0.4*(−0.9,1.8)	2000–2003	0.8(−2.1,3.8)
Trend4	2011–2014	4.7*(1.4,8.1)	2004–2009	4.2*(4.1,4.4)	2003–2011	4.5*(4.1,4.9)
Trend5	2014–2017	0.6(−1.6,2.8)	2009–2017	1.3*(−0.3,2.9)	2011–2017	0.8*(0.1,1.4)
AAPC *	1990–2017	4.5*(4.0,5.0)	1990–2017	1.8*(1.6,2.0)	1990–2017	2.4*(1.9,2.8)
The U.S.						
Trend1	1990–1995	4.8*(4.1,5.5)	1990–1995	3.6*(3.0,4.3)	1990–1993	3.3*(1.8,4.9)
Trend2	1995–2001	1.4*(0.8,2.1)	1995–1998	1.3(−1,4,4.1)	1993–2002	2.0*(1.7,2.3)
Trend3	2001–2004	−1.0(−3.6,1.7)	1998–2001	4.3*(1.6,7.1)	2002–2005	−0.2(−2.9,2.6)
Trend4	2004–2009	1.0*(0.1,1.8)	2001–2009	1.3*(0.9,1.6)	2005–2009	1.5*(0.0,3.0)
Trend5	2009–2017	0.5*(0.1,0.9)	2009–2017	−0.5*(− 0.8,-0.1)	2009–2017	0.2(− 0.2,0.5)
AAPC *	1990–2017	1.4*(1.0,1.8)	1990–2017	1.5*(1.1,1.9)	1990–2017	1.3*(0.9,1.7)

Note: * *APC* annual percentage change; *AAPC* average annual percent change; *CI* confidence interval; *Significantly different from 0 at alpha = 0.05 (*p* < 0.05). There are four joinpoints for each model. The estimate of the comparability ratio (CR) from JP-Jump Model for China men is 1.01(0.9, 1.03) with standard error = 0.011. The CR is 0.97(0.96, 0.99) for China women with standard error = 0.007. The CR is 1.03(0.99, 1.07) for U.S. men with standard error = 0.017. The CR is 0.97(0.96, 1.02) for U.S. women with standard error = 0.015

### The age, period, and cohort effects on TC incidence

**Fig. 2:**
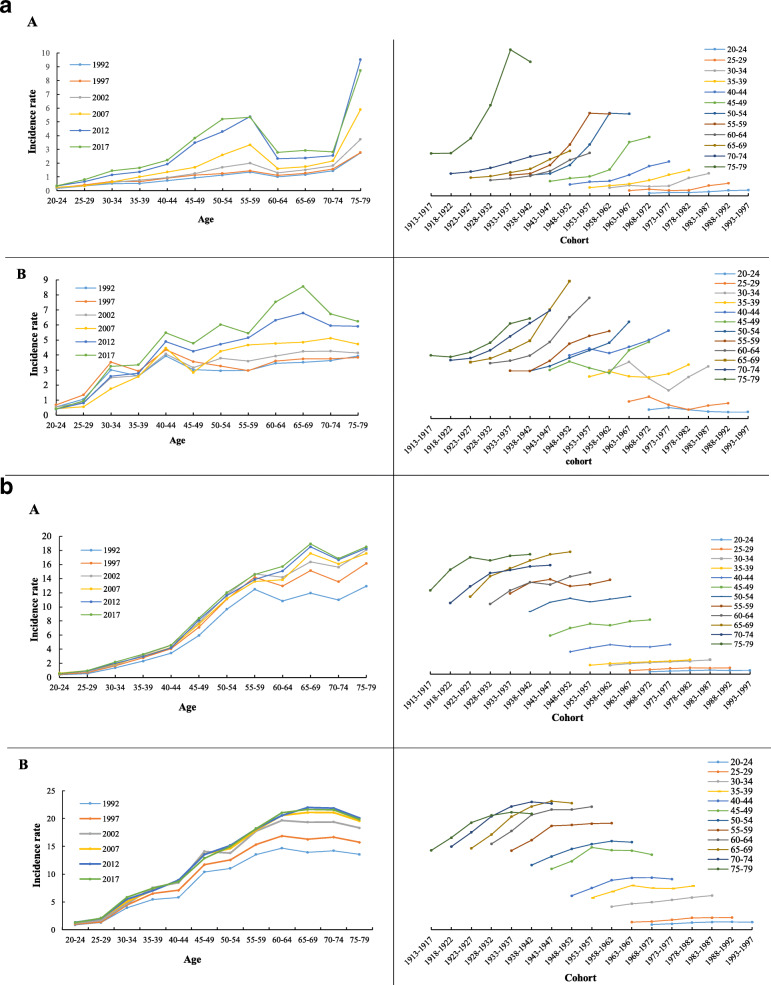
**a**. Age-standard incidence rates (ASIR) of thyroid cancer across ages and birth cohorts by periods and age groups among (A) men and (B) women in China. **b**. Age-standard incidence rates (ASIR) of thyroid cancer across ages and birth cohort by period and age group among (A) men and (B) women in U.S

We reported the incidence of age-specific TC in Fig. 2a and Fig. 2b. The incidence had different peaks in different age groups, as given below: Chinese men have two peak periods, 55 to 59 years and 75 to 79 years. The TC incidence also had a turning point in birth cohorts. The incidence of Chinese women has continued to rise from the age of 20 to 70, and peaked at the age of 65–69. The cohort of each age group showed that the incidence of the earlier period was lower than the incidence of later. In the U.S., the peak incidence of men and women is 65 to 69 age group. There is no turning point for birth cohorts of every age. Among men and women of all ages in China and the U. S., men who were older had in general higher TC incidence than younger men. Similarly for women, those born later had lower TC incidence and older women had a higher incidence.

The APC model was listed for men and women separately. The relative risk of TC caused by age, period, and cohort is given in Table 2. The APC-IE method showed the estimated coefficients of age, period, and cohort effects (Table 3).

**Table 2:** Thyroid cancer incidence relative risks (RR) of 95% confidence interval (CI) due to age period cohort Thyroid cancer incidence relative risks (RR) and 95% confidence interval (CI) by age period cohort

Variables	The U.S.(RR 95%CI)		China (RR 95%CI)	
	Men	Women	Men	Women
Age				
20–24	1.00	1.00	1.00	1.00
25–29	1.67 (2.77,1.01)	1.52 (2.04,1.13)	1.46 (1.91,1.12)	1.41 (1.67,1.20)
30–34	2.68 (5.43,1.32)	3.96 (6.84,2,30)	3.00 (4.83,1.87)	3.77 (5.34,2.66)
35–39	3.02 (6.44,1.41)	3.64 (6.32,2.10)	4.52 (7.99,2.56)	4.98 (7.35,3.38)
40–44	3.80 (8.87,1.63)	5.29 (10.01,2.80)	5.97 (11.25,3.17)	5.42 (8.17,3.59)
45–49	5.45 (14.19,2.09)	3.91 (7.22,2.12)	9.71 (19.95,4.73)	7.96 (12.66,5.01)
50–54	7.08 (19.76,2.53)	4.42 (8.44,2.32)	13.19 (28.73,6.06)	7.93 (12.84,4.90)
55–59	8.29 (24.36,2.82)	4.40 (8.45.2.29)	14.89 (33.73,6.57)	9.10 (15.14,5.47)
60–64	4.33 (11.53,1.63)	5.19 (10.30,2.62)	13.44 (30.94,5.84)	9.60 (16.24,5.68)
65–69	4.65 (12.86,1.68)	5.70 (11.41,2.85)	14.71 (34.40,6.29)	9.14 (15.50,5.39)
70–74	5.06 (14.43,1.78)	5.54 (10.94,2.80)	12.40 (28.48,5.40)	8.67 (14.63,5.13)
75–79	12.58 (39.74,3.98)	5.44 (10.42,2.85)	13.04 (29.09,5.84)	7.68 (12.64,4.67)
Period				
1992	1.00	1.00	1.00	1.00
1997	1.15 (1.21,1.10)	1.10 (1.12,1.08)	1.30 (1.36,1.24)	1.23 (1.26,1.19)
2002	1.40 (1.53,1.28)	1.11 (1.14,1.09)	1.54 (1.65,1.44)	1.51 (1.57,1.45)
2007	2.00 (2.31,1.73)	1.23 (1.27,1.19)	1.66 (1.77,1.55)	1.68 (1.75,1.61)
2012	3.28 (4.03,2.68)	1.56 (1.65,1.48)	1.89 (1.99,1.78)	1.83 (1.90,1.76)
2017	3.81 (4.70,3.09)	1.90 (2.02,1.79)	2.13 (2.22,2.05)	1.95 (2.01,1.89)
Cohort				
1913–1917	1.00	1.00	1.00	1.00
1918–1922	0.99 (1.24,0.79)	0.90 (1.15,0.71)	0.93 (1.09,0.80)	0.94 (1.09,0.80)
1923–1927	1.05 (1.56,0.71)	0.89 (1.27,0.62)	0.86 (1.10,0.68)	0.88 (1.11,0.71)
1928–1932	1.09 (1.83,0.65)	0.92 (1.42,0.60)	0.81 (1.08,0.61)	0.85 (1.10,0.65)
1933–1937	1.01 (1.89,0.53)	0.95 (1.54,0.59)	0.77 (1.05,0.56)	0.81 (1.09,0.61)
1938–1942	0.80 (1.56,0.41)	0.91 (1.52,0.54)	0.71 (0.98,0.51)	0.77 (1.05,0.57)
1943–1947	0.73 (1.25,0.43)	0.99 (1.63,0.60)	0.64 (0.86,0.48)	0.73 (0.97,0.55)
1948–1952	0.81 (1.36,0.49)	1.10 (1.80,0.67)	0.58 (0.75,0.46)	0.67 (0.86,0.51)
1953–1957	0.84 (1.38,0.52)	1.04 (1.65,0.66)	0.52 (0.64,0.43)	0.63 (0.80,0.50)
1958–1962	0.80 (1.28,0.50)	0.94 (1.44,0.61)	0.47 (0.55,0.41)	0.59 (0.72,0.48)
1963–1967	0.88 (1.36,0.57)	0.98 (1.49,0.65)	0.44 (0.48,0.40)	0.54 (0.65,0.46)
1968–1972	0.79 (1.13,0.56)	0.84 (1.22,0.58)	0.40 (0.40,0.39)	0.48 (0.55,0.42)
1973–1977	0.66 (0.81,0.53)	0.68 (0.92,0.51)	0.36 (0.34,0.39)	0.45 (0.49,0.42)
1978–1982	0.62 (0.68,0.56)	0.61 (0.72,0.51)	0.36 (0.30,0.43)	0.45 (0.45,0.44)
1983–1987	0.61 (0.58,0.64)	0.56 (0.58,0.54)	0.35 (0.24,0.51)	0.45 (0.41,0.49)
1988–1992	0.55 (0.33,0.90)	0.42 (0.24,0.73)	0.31 (0.13,0.70)	0.42 (0.27,0.65)
1993–1997	0.43 (0.05,4.00)	0.30 (0.04,2.43)	0.27 (0.03,2.09)	0.37 (0.11,1.29)
AIC	3.19	3,91	4.48	4.92
BIC	−170.03	− 169.73	−170.30	−170.68
Deviance	1.04	1.34	0.77	0.39

Note: *AIC* Akaike’s information criterion; *BIC* Bayesian information criterion

**Table 3:** Thyroid cancer incidence rates estimated coefficients and 95% confidence interval (CI) for the age, period and cohort effects

Variables	The U.S.(Coef,95%CI)		China (Coef,95%CI)	
	Men	Women	Men	Women
Age				
20–24	−1.41(−3.02,0.21)	− 1.32(− 2.39,-0.26)	−1.90(− 2.97,-0.81)	−1.67(−2.40,-0.94)
25–29	− 0.89(− 2.00,0.22)	− 0.91(− 1.68,-0.14)	−1.51(− 2.33,-0,69)	−1.32(− 1.88,-0.76)
30–34	− 0.42(− 1.33,0.49)	0.05(− 0.47,0.57)	− 0.79(− 1.40,-0.19)	− 0.34(− 0.72,0.04)
35–39	− 0.30(− 1.16, 0.56)	− 0.03(− 0.54,0.48)	− 0.38(− 0.89,0.13)	− 0.06(− 0.40,0.28)
40–44	− 0.07(− 0.84, 0.70)	0.34(− 0.08,0.77)	− 0.11(− 0.55,-0.34)	0.02(− 0.29,0.34)
45–49	0.29(− 0.37,0.95)	0.04(− 0.41,0.49)	0.38 (0.02,0.74)	0.41 (0.14,0.67)
50–54	0.55(− 0.04, 1.14)	0.16(− 0.26,0.58)	0.68 (0.39,0.99)	0.40 (0.16,0.65)
55–59	0.71 (0.17,1.25)	0.16(− 0.25,0.57)	0.91 (0.55,1.07)	0.54 (0.32,0.76)
60–64	0.06(− 0.58,0.70)	0.32(− 0.06,0.70)	0.71 (0.46,0.95)	0.60 (0.39,0.80)
65–69	0.13(− 0.47,0.73)	0.42 (0.05,0.79)	0.80 (0.57,1.03)	0.55 (0.35,0.75)
70–74	0.22(− 0.35,0.79)	0.39 (0.00,0.77)	0.63 (0.38,0.87)	0.49 (0.29,0.70)
75–79	1.13 (0.66,1.59)	0.37(− 0.04,0.79)	0.68 (0.40,0.95)	0.37 (0.14,0.60)
Period				
1992	−0.62(− 1.18,-0.05)	− 0.25(− 0.57,0.07)	−0.43(− 0.68,-0.18)	−0.40(− 0.59,-0.21)
1997	− 0.48(− 0.99,0.04)	−0.16(− 0.46,0.14)	−0.17(− 0.37,0.03)	−0.20(− 0.37,-0.03)
2002	− 0.28(− 0.75,0.19)	−0.14(− 0.44,0.15)	0.00(− 0.18,0.18)	0.01(− 0.14,0.16)
2007	0.08(− 0.34,0.50)	−0.04(− 0.32,0.24)	0.07(− 0.11,0.25)	0.12(− 0.03,0.27)
2012	0.57 (0.21,0.93)	0.20(− 0.07,0.46)	0.20 (0.01,0.39)	0.20 (0.05,0.36)
2017	0.72 (0.37,1.08)	0.39 (0.13,0.65)	0.33 (0.12,0.53)	0.27 (0.10,0.43)
Cohort				
1913–1917	0.25(−0.96,1.46)	0.24(−0.71,1.19)	0.63 (0.04,1.23)	0.48(−0.05,1.01)
1918–1922	0.23(−0.75,1.22)	0.14(− 0.57,0.84)	0.56 (0.13,1.00)	0.41 (0.04,0.79)
1923–1927	0.30(−0.51,1.12)	0.12(−0.47,0.72)	0.48 (0.13,0.84)	0.35 (0,05,0.66)
1928–1932	0.33(−0.36,1.02)	0.16(−0.36,0.68)	0.43 (0.12,0.73)	0.31 (0.05,0.57)
1933–1937	0.25(−0.32,0.83)	0.19(−0.27,0.66)	0.37 (0.09,0.65)	0.27 (0.03,0.51)
1938–1942	0.02(−0.52,0.57)	0.15(−0.29,0.58)	0.29 (0.02,0.56)	0.22(−0.01,0.45)
1943–1947	−0.06(− 0.74,0.61)	0.23(− 0.22,0.68)	0.20(− 0.11,0.50)	0.17(− 0.08,0.41)
1948–1952	0.04(− 0.66,0.74)	0.34(− 0.12,0.79)	0.10(−0.25,0.44)	0.07(− 0.20,0.34)
1953–1957	0.08(− 0.64,0.79)	0.28(− 0.21,0.77)	−0.02(− 0.41,0.38)	0.02(− 0.28,0.31)
1958–1962	0.02(− 0.71,0.75)	0.18(− 0.34,0.70)	−0.12(− 0.56,0.32)	−0.06(− 0.38,0.27)
1963–1967	0.12(− 0.66,0,89)	0.22(− 0.31,0.76)	−0.19(− 0.70,0.31)	−0.13(− 0.49,0.23)
1968–1972	0.01(− 0.84,0.87)	0.07(− 0.51,0.65)	−0.29(− 0.87,0.28)	− 025(− 0.65,0.14)
1973–1977	−0.17(−1.17,0,83)	−0.14(− 0.80,0.52)	−0.38(− 1.05,0.30)	−0.31(− 0.77,0.14)
1978–1982	−0.24(− 1.35,0.88)	−0.26(− 1.04,0.52)	−0.40(− 1.18,0.38)	−0.33(− 0.84,0.18)
1983–1987	−0.24(− 1.50,1.01)	−0.34(− 1.26,0.58)	−0.42(− 1.39,0.55)	−0.33(− 0.95,0.30)
1988–1992	−0.35(− 2.06,1.35)	−0.62(− 2.13,0.88)	−0.55 (2.00,0.87)	− 0.39(− 1.35,0.57)
1993–1997	−0.59(− 4.02,2.84)	−0.96(− 4.00,2.08)	−0.69(− 3.34,1.97)	−0.50(− 2.27,1.26)

### Age-period-cohort analysis

#### Age effect

**Fig. 3:**
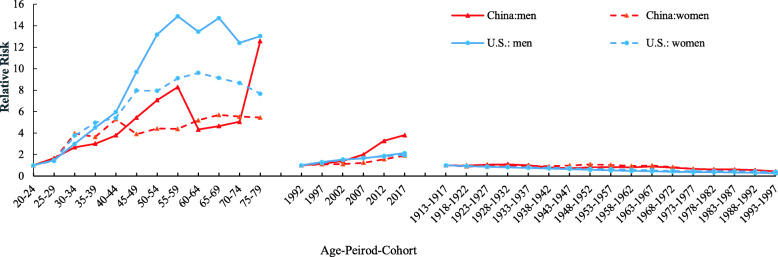
Thyroid cancer incidence relative risks due to age; period; and cohort effects for men and women using APC model

After controlling the effects of the period and cohort, we found that the age effect was significantly related to the incidence of TC (Fig. 3). In general, except for the 55–59 age group in China and a slowly decrease for 55–59 and 65–69 age groups in the U. S, the risk ratios (RRs) for men have increased, and there is a distinct peak for men in the age effect. The TC incidence of China and U.S. in the 75–79 age group was about 12.58 and 13.04 time higher than that of the 20–24 age group for men. The risk of TC incidence was about 5.44 and 7.68 time higher than that of the 20–24 age group for women, which indicated that the incidence of TC increased slowly with advancing age. However, the most prominent part is that China RRs has a rapid downward trend in the 60–64 age group. Overall, the RRs of men and women in the U.S. were higher than that in China.

#### Period effect

In addition to the age effect on the incidence of disease, we observed that time is highly dependent on the disease. Periodic RRs of TC incidence in China and the U.S. are plotted in Fig. 3. Generally, the risk of TC incidence substantially increased with the period for both genders in China and the U.S. Compared to the period RRs in 1992, the RRs of China incidence in 2017 in men and women was 3.81 and 1.95, respectively. Among the periods for Chinese men, the RRs of incidence markedly increased by 39.02% from 2007 to 2012. For women, the period effects of TC incidence were relatively stable in the U.S.

#### Cohort effect

We demonstrated the cohort RRs of TC incidence in China and the U.S. in Fig. 3. The curves displayed a downward trend for both genders. In general, it can be seen that the cohort effect is related to the cohort RRs of TC incidence in China, people born after 1973 have reduced the risk of TC, and it can be estimated that this effect is significant from the confidence interval. Chinese men had risen quickly since 1913, and then displayed a sharp decline from 1932 to 1947. The associated curve of Chinese women showed a gradual increase to the highest point, then had a gentle decline for the 1962 birth cohort, and finally displayed a sharp decline in the birth cohort. Compared to China, the patterns were found to have different behavior in U.S. Later birth cohorts showed lower RRs than early birth cohorts did in the U.S. The cohort RRs of TC incidence in the U.S. showed a declining trend among the cohorts for both genders; while in general, men had always been lower than women in all birth cohorts had.

## Discussion

In our study, we analyzed the temporal trends and the changes in the incidence of TC in China and the U. S. from 1990 to 2017. Among Chinese men, 2006–2011 was a surprisingly “Joinpoint”. The reason for the significant upward is most likely the frequency of medical diagnostic and therapeutic nuclear medicine resulting in an increased incidence of TC [27]. In recent years, the incidence rate of the two countries has been steadily slowing down. It can be seen that China and the U.S. have implemented policies to improve cancer screening. We also found that from 1992 to 2017, the incidence of ASIR of TC in China and in the U. S. was higher in women than in men and still at a relatively higher level. We observed the incidence of TC vary by sex, and estrogen levels of women may be one of the risk factors for TC [28]. In addition, a study is similar to our results. The long-term trend for women is greater, while the age-related trend for men is greater [29]. Otherwise, Joinpoint regression analysis presented incidence rates increased in both men and women from 1992 to 2017, while incidence rates obviously increased in Chinese men mainly from 2006 to 2011. Because the important interaction between age, period and cohort effect, we use an age-period-cohort model and IEalgorithm to explore the impact of these factors on the incidence of TC. Therefore, it is necessary to further analyze the causes and differences of these trends in the model, and further explore the risk factors leading to the occurrence of TC disease. In general, the incidence of TC increases with age and period, especially the 40–75 age groups of men in the U.S. The men showed a significant upward trend from 2007 to 2017, but decreased with the birth cohort in China.

Based on our findings, the incidence of TC increased with the age of men and women, and China’s aging may exacerbate this situation [30]. Chinese men have a higher risk of TC in the 40–55 age group, probably because residents between the ages of 40–59 paid more attention to physical examination, and the detection rate in cities are higher than in rural areas [31]. The rapid rise in age RRs of Chinese men for the 70–79 age group is due to the most variability of cancer prognosis in the old ages and the highest risk of TC treatment. Among the female population in China, the fastest growth was in women of childbearing age, which is consistent with the results of other countries [15]. The rapid increase RRs of 20–30 age groups of women may be an annual obstetric and gynecological examination during reproduction in China. The risk of TC in women increases during puberty, but decreases after menopause. Among young women, the incidence of TC ranks first among malignant tumors. It is suggested that hormone factors are involved in TC, and estrogen increases thyroid growth [32]. According to our results, old people had a higher risk of TC than young. The age effects may increase the risk of illness in the elder people. In addition, increased complications of TC are existed in older TC patients than in younger patients. China’s aging is growing faster than the U.S., the trend of population growth and aging is increasing in China [33]. Therefore, we may pay more attention to prevention and control the occurrence of the TC in the old age people.

The RRs of TC in women increased rapidly with the age of before 45-year-old age group in the U.S. However, the RRs of TC decreased rapidly after the 60-year-old, which may indicate that estrogen levels play a role in the development of TC. There were obvious gender differences in the incidence of TC. Women were 2–3 times more likely than men for TC incidence from 1992 to 2017. It further indicates that TC may be related to estrogen [34]. The age RRs of TC for U.S. men increased slowly from 20 to 49 years but increased rapidly after 50 years of age. Therefore, among men over 50 years old, the diagnosis rate of papillary TC is high, but over diagnosis is rare among men between 20 and 49 years old in the U. S. Among patients with TC in the U. S., the majority of patients in diagnosis were 45–49 age groups for women and 55–59 age groups for men [35]. The difference in age and gender may be that middle-aged women use more health care services than men. This difference is also a result of reproductive activity and menopause, leading to earlier diagnosis [7, 36]. Moreover, compared with women, men tend to pay more attention to their health when they get old. According to a study, the increased TC incidence rates have been reported among young people and adults in the U.S. [37], which shows that some risk factors, such as high body mass index (BMI), [38] ionizing radiation, [39] may have contributed to increasing TC incidence rate.

The period effect means that unique medical technology, diagnostic methods, economic and cultural changes in a specific period can increase the risk of TC. Environmental factors are closely related to TC, that is, exposure to ionizing radiation is one of the TC risk factors [40, 41]. On the whole, the period RRs of men and women in China is on the rise. From 1992 to 2008, the frequency of medical diagnostic and therapeutic nuclear medicine in China has been increasing, resulting in a significant increase in the annual personal radiation dose [42]. It is not difficult to find that the period effect of men and women after 2007 in China has a clear rising trend. Among them, men showed a slow upward trend after 2012, while women increased significantly from 2007 to 2017. A previous study conducted in China reported that in 2008, about 17% of people over the age of 15 had regular physical examinations, 68% had a physical examination every 7–12 months, and one-third of the population conducted X-rays. More frequent nuclear medical examinations may lead to increasing radiation exposure, resulting in an increased incidence of TC [27]. With the acceleration of China’s industrialization process, the exposure to risk factors is becoming more and more obvious [43]. From 1992 to 2017, men’s carcinogens increased significantly. The rising incidence of men may be due to cancer caused by occupational diseases [18]. Over the past 25 years, environmental exposure caused by industrial activities of industrial density may have affected the incidence of TC, which is an occupational carcinogen. TC is considered a cancer that can be affected by occupational exposure. In addition, the GBD study also showed that cancer death and disability-adjusted life years (DALYs) due to occupational risks began to increase around 2007. In fact, occupational exposure has increased since 2007 [44]. Especially, male workers who are exposed to certain solvents and pesticides have an increased risk of developing thyroid tumors. Period RRs showed a remarkable increasing trend of Chinese men, which may be caused by occupational carcinogens. Therefore, it is necessary to minimize the exposure of men to occupational carcinogens in China [45].

In the U. S., men and women period effects have a similar upward trend. TC is one of the ten most common cancers in the U.S. From 1990 to 2017, the incidence of TC has been increasing at a higher rate than any other malignant tumor. The survey data of the U.S. from 2003 to 2009,revealed that the 5-year survival rate of TC reached up to 98.2% [46]. The steady growth trend of men and women may be due to the rise in the economy of the U.S. The increased incidence of TC was caused by over diagnosis from 1990 to 2000 [35]. The socio-economic progress of the U.S. is rapidly increased. In general, people with higher socioeconomic status are more likely to access health care services, and have increased contact with the health care system, leading to an increased risk of over diagnosis. A positive correlation is existed between socioeconomic status and TC risk [47].

The cohort effect represents the impact of early life economic level, living habits and environmental factors on the risk of TC. Early in life exposure to adverse risk factors may affect future life [48]. Men and women simultaneously increased the risk of morbidity in the cohort effect. This may be linked to China’s economic development and related environmental and cultural changes. One stage was from 1943 to 1947 to 1948–1952, which was considered the period of the War of Resistance against Japan and the period of the National Liberation War. After a long period of war, the living environment has deteriorated and health problems have become more difficult to defend. In addition, during the periods of 1958–1962 and 1963–1967, China experienced a series of social and economic system changes, and the three-year natural disasters from 1959 to 1961, due to the sacrifice of agriculture and industrial development policies, this led to a nationwide food shortage crisis. Therefore, health risk factors have increased significantly [49]. At the same time, this disaster has also aggravated the spread of some infectious diseases, which has adverse effects on people’s health and industrial and agricultural production. In terms of gender differences in China, we found that women in the early cohort had a lower risk of incidence than men incidence. After the birth cohort in 1933–1937, the risk of women in the incidence began to be higher than that of men, and in the birth cohort after 1978–1982, the risk of women began to be lower than that of men. Obviously, women greatly reduced the risk of the birth cohort after 1952. It may be due to the establishment of the employee medical security system in China and the improvement of the Chinese medical system at that time [50, 51]. During this period, China launched an important patriotic public health campaign, which greatly improved the health of residents and decreased the risk of developing TC by carrying out pest control, health education and health promotion [52].. The increased risk of incidence in later-born men is related to social disintegration and later-born men are more likely to be exposed to smoking, drinking, and other risk factors that lead to higher risk levels. Complex interactions between these risk factors may lead to increasing TC risk of Chinese men [53]. In the U. S., ultraviolet radiation may be one of the risk factors for TC. Because, since 1960s, the availability of medical services in the U.S., new equipment and complex diagnostic tests has increased, the number of X-rays for medical examinations and care has dramatically increased [54]. We found that people over the age of 40 are more likely to be exposed to ultraviolet radiation than younger people. It reflects that the cohort effect of the TC is on declining trend [55]. During 1913–1917 and 1993–1997, the incidence of TC for men and women showed a continuous downward trend in the U.S. The possible reason is that the recently born cohort is well-educated and gains a better understanding of health and disease prevention [56]. People born after 1973 have a reduced risk of TC incidence in China and the U.S. Previous studies can be seen that under the influence of changes in medical practice and improved diagnostic technology of TC, with the introduction of thyroid ultrasound and the emergence of needle aspiration technology in the 1980s, the sensitivity of diagnosis and detection opportunities have improved [29, 57].

Since the 1990s, the incidence of TC has been growing faster than other types of cancer in the U. S as far as China is worried, TC ranks seventh in the incidence of malignant tumors. China holds the largest number of deaths from TC in the world. The results of recent studies show that the countries with the highest incidence of TC are China and the U.S., and the global rise of TC incidence is related to geographic factors. The incidence of TC in China is also significantly different based on gender and age. Therefore, more evidence-based studies are required to confirm the existing findings regrading TC trends in these countries [1, 58, 59].

### Limitations

We acknowledge that our research has certain limitations.. Firstly, the data used in this study are based on the latest data from GBD 2017, which uses incidence data from the Cancer Registry. The accuracy of the diagnosis is still biased, which will lead to the problem of decreased data accuracy. Secondly, our research on TC had no specific cancer subdivisions or in-depth research on the factors that lead to changes in TC, so we cannot make causal inferences. Despite its limitations, our study is still an indispensable national study, which aims to compare the trends and changes in the incidence of TC in China and the U.S.

## Conclusion

In summary, the incidence of TC in men and women in China and in the U.S. from 1990 to 2017 was attributed to the following factors: over diagnosis and screening, a significant increase in ionizing radiation, and the effect of estrogen on TC. We observe that period effect has a growth trend in China, while it is steadily increasing in the U. S. The cohort effect shows a downward trend, but China’s fluctuations are observable. The age effect may be a critical factor affecting the incidence of TC, but the peaks of different age groups in both China and the U. S are different. China’s population growth and aging have an impact on the incidence of TC. Therefore, it is necessary to provide reasonable health services and screening to reduce ionizing radiation. At the same time, we need to strengthen the prevention and protection of TC for women.

## Data Availability

The dataset analyzed during the current study are available in the Institute for Health Metrics and Evaluation (IHME): http://ghdx.healthdata.org/gbd-results-tool.

## Abbreviations

TC: Thyroid cancer
IHME: Institute for health metrics and evaluation
ICD: International Classification of Diseases
IE: Intrinsic estimator
BMI: Body mass index
CR: Comparability ratio
